# New cell delivery system CellSaic with adipose-derived stromal cells promotes functional angiogenesis in critical limb ischemia model mice

**DOI:** 10.1007/s10047-021-01254-8

**Published:** 2021-03-03

**Authors:** Hideki Tanioka, Shigeru Miyagawa, Daisuke Mori, Ken-ichi Watanabe, Takayoshi Ueno, Koichi Toda, Takashi Shibuya, Toru Kuratani, Yoshiki Sawa

**Affiliations:** Department of Cardiovascular Surgery, Osaka University, 2-15 Yamadaoka, Suita, Osaka Japan

**Keywords:** Critical limb ischemia, Angiogenesis, Adipose-derived stromal cell, Engraftment rate

## Abstract

Current therapies for patients with critical limb ischemia have not reduced amputation risk owing to poor cell engraftment. The recombinant peptide Cellnest increases the engraftment rate of administered cells by forming a complex with the cells (CellSaic). We hypothesized that CellSaic containing adipose-derived stromal cells (ADSCs) could improve lower limb blood flow better than ADSCs alone, resulting in better transplanted cell engraftment. ADSCs were extracted from 8-week-old C57BL/6N mice. Thirty-two critical limb ischemia model mice were established by ligating femoral arteries. They were divided into CellSaic (*n* = 11), ADSC (*n* = 10), saline (*n* = 9), and Cellnest (*n* = 9) groups. Blood flow rate (affected side blood flow / healthy side blood flow × 100%) was evaluated using a laser Doppler blood flow meter every week. Mice were euthanized on day 28 for histological evaluation. Compared with the ADSC group (54.5 ± 17.2%), treated side blood flow rate of the CellSaic group (78.0 ± 24.9%) showed significant improvement on day 28 after administration (*p* < 0.05). CD31 staining showed significantly higher number of capillary vessels in the CellSaic group (53.0 ± 8.9 cells/mm^3^) than in the ADSC group (43.0 ± 6.8 cells/mm^3^) (*p* < 0.05). Fluorescent staining showed significantly higher number of arterioles containing both CD31 and αSMA double-positive cells in the CellSaic group than in the ADSC group (*p* < 0.05). CellSaic containing ADSCs exhibited superiority to ADSC transplantation alone in promoting functional angiogenesis, suggesting its potential in improving clinical outcomes of angiogenic therapy for ischemic limbs.

## Introduction

Approximately, 30% patients with critical limb ischemia (CLI) lose their limbs, and approximately 25% die within 1 year despite several treatment options, such as surgical revascularization, percutaneous transluminal angioplasty, and drug therapy [1]. New angiogenesis therapies for CLI, such as gene and cell therapy, are expected to overcome these clinical problems. Many types of cells have been used for angiogenesis therapy, but clinical results, such as increase in the salvage rate of ischemic limbs, are limited because of the poor engraftment rate of transplanted cells [2]. Therefore, improving the engraftment rate of transplanted cells is crucial for achieving a clinically meaningful endpoint [2].

It has been reported that the recombinant peptide Cellnest increases the engraftment rate of administered cells by forming a complex with the cells, named CellSaic. Cellnest contains the cell adhesion RGD sequence of the human type I collagen α chain and can be used as a scaffold material for cells. CellSaic has been used to transplant pancreatic islet cells and has shown improvements in engraftment rate because of adequate intercellular spaces resulting in the migration of nutrient blood vessels [3].

In this study, we hypothesized that compared with adipose-derived stromal cells (ADSCs) alone, CellSaic containing ADSCs improves lower limb blood flow.

## Materials and methods

### Ethical approval

The Institute of Experimental Animal Science at Osaka University approved this research (approval number: 30-097-000). Institutional guidelines for the care and use of laboratory animals were observed.

### Animals

Mice were the only animals used in this experiment. An inhalation anesthetic (isoflurane) was used initially and intraperitoneal anesthetics (midazolam and medetomidine) were added subsequently to relieve pain during treatment. Euthanasia was achieved through inhalation anesthetic (isoflurane) overdose.

### Cell culture

ADSCs were isolated from the inguinal adipose tissue of ten 8-week-old male C57BL/6N mice according to the method described by Nakagami et al. [4] with minor modifications. The inguinal adipose tissue samples were treated with 0.1% collagenase and cultured in Dulbecco’s modified Eagle’s medium (DMEM) supplemented with 10% fetal bovine serum and 1% penicillin–streptomycin mixture. We used ADSCs at passage 5 in this experiment. Similarly, ADSCs were isolated from ten 8-week-old male C57BL/6-Tg (CAG-EGFP) mice to evaluate the engraftment rate of these cells. The identity of ADSCs was confirmed through the evaluation of various cell surface markers using flow cytometry. ADSCs were mechanically dissociated and resuspended in fluorescence-activated cell sorting (FACS) staining buffer (phosphate-buffered saline supplemented with 5% fetal bovine serum). The antibodies used for FACS analysis included rat anti-mouse CD44, CD73, CD90, and Sca-1 (BD Biosciences, San Jose, CA, USA) conjugated to fluorescein isothiocyanate or phycoerythrin. The cells were stained for 30 min at room temperature, washed, and examined using the BD FACSVerse™ Flow Cytometer (BD Biosciences). The data were analyzed using BD FACSDiva™ (BD Biosciences).

### CellSaic synthesis

CellSaic platforms containing ADSCs were prepared by mixing ADSCs (2.0 × 10^6^ cells/mL) and cellnest (2 mg) in 20 mL of DMEM. This mixture was seeded on the PrimeSurface 96U plate (Sumitomo Bakelite Co. Ltd., Tokyo, Japan) in 200 μL wells. Each well contained 2.0 × 10^4^ cells and 0.02 mg cellnest. Each plate was centrifuged using a tabletop plate centrifuge (600×*g* for 5 min) and then incubated for 24 h in a CO_2_ incubator.

### Establishment of the mouse hindlimb ischemic model and evaluation of the blood flow rate

We established 29 mouse models of critical limb ischemia (8-week-old male C57BL/6N mice) by ligating the femoral arteries. The mice were divided into the CellSaic (*n* = 11), ADSC (*n* = 10), saline (*n* = 9), and Cellnest (*n* = 9) groups. The mice were anesthetized with midazolam (5 mg/kg) and medetomidine (0.5 mg/kg) via intraperitoneal administration. The femoral arteries were ligated at the level of the inguinal ligament and knee joint and removed. After 7 days, the blood flow rate was evaluated using a laser Doppler imaging (LDI) flowmeter (Moor Instruments, Devon, UK), and mice whose affected side blood flow rate recovered to 10%–20% of the healthy side blood flow rate were defined as the critical limb ischemia models. CellSaic, ADSCs, saline, and Cellnest were administered to the critical limb ischemia model mice. The blood flow rate (affected side blood flow/healthy side blood flow × 100%) was measured with an LDI flowmeter every week until the 28th day after administration. The mice were euthanized on the 28th day to evaluate femoral muscle tissue histology and to collect samples for polymerase chain reaction (PCR).

### Histological analysis

The femoral muscle tissue was formalin-fixed, paraffin-embedded, and cut into 5-μm-thick sections using a microtome for histological analyses. Using immunohistochemistry, the sections were labeled with a polyclonal anti-antibody (anti-CD31, Abcam, ab28364; anti-αSMA, Dako, M0851) and visualized using the LSAB kit (Dako, Glostrup, Denmark, K0690), which is an automated immunostaining system based on the Lepto-streptavidin–biotin–peroxidase method. The sections were then stained with the corresponding secondary antibodies (Alexa Fluor 555 or Alexa Fluor 488, Molecular Probes, Eugene, OR, USA). The number of CD31-positive cells was evaluated by averaging five visual fields of five sections using an optical microscope (Keyence, Osaka, Japan). The percentage of double-positive cells was calculated by averaging five visual fields of five sections based on fluorescence staining using a confocal microscope (Olympus, Tokyo, Japan).

### Analysis of angiogenic factor by PCR

RNA was isolated from femoral muscle tissue using an RNeasy Micro kit (Qiagen, Valencia, CA, USA), according to the manufacturer’s instructions. RNA was quantified using the NanoDrop ND-1000 spectrophotometer (NanoDrop Technologies, Inc., Wilmington, DE, USA). cDNA was obtained by the reverse transcription of RNA using the SuperScript™ VILO™ cDNA Synthesis Kit (Invitrogen, Carlsbad, CA, USA), according to the manufacturer’s instructions. Angiogenic factors such as vascular endothelial growth factor (VEGF) and hepatocyte growth factor (HGF) in the muscle tissues were analyzed by reverse-transcription PCR using TaqMan Fast Advanced Master Mix and QuantStudio (Thermo Fisher Scientific, Waltham, MA, USA). Expression of each mRNA was normalized to that of glyceraldehyde-3-phosphate dehydrogenase (Applied Biosystems): *GAPDH* (Mm99999915_g1). The following genes were analyzed using the TaqMan gene expression assay (Applied Biosystems): *VEGF* (Mm01281449_m1) and *HGF* (Mm01135184_m1).

### Engraftment rate of ADSCs

We established 19 mouse models of critical limb ischemia (8-week-old male C57BL/6N mice) by ligating the femoral arteries. The mice were divided into the CellSaic (containing ADSCs isolated from GFP mice) (*n* = 10) and ADSC (ADSCs isolated from GFP mice) (*n* = 9) groups. CellSaic and ADSCs were administered to the critical limb ischemia model mice. The mice were euthanized on the 28th day after administration to evaluate the engraftment rate based on femoral muscle tissue histology. The muscle tissue was stained with monoclonal anti-αSMA (Dako, M0851) as the primary antibody and then the corresponding secondary antibodies (Alexa Fluor 555, Molecular Probes, Eugene, OR, USA). The number of GFP-positive cells was enumerated.

### Statistical analysis

Data were analyzed using JMP Pro 13 (SAS Institute, Cary, NC, USA). All data, except PCR results, are presented as mean and standard deviation. PCR results are presented as mean and standard error. Differences between groups were determined using Student’s t test. Differences were considered statistically significant at *p* < 0.05.

## Results

### Properties of ADSCs

**Fig. 1:**
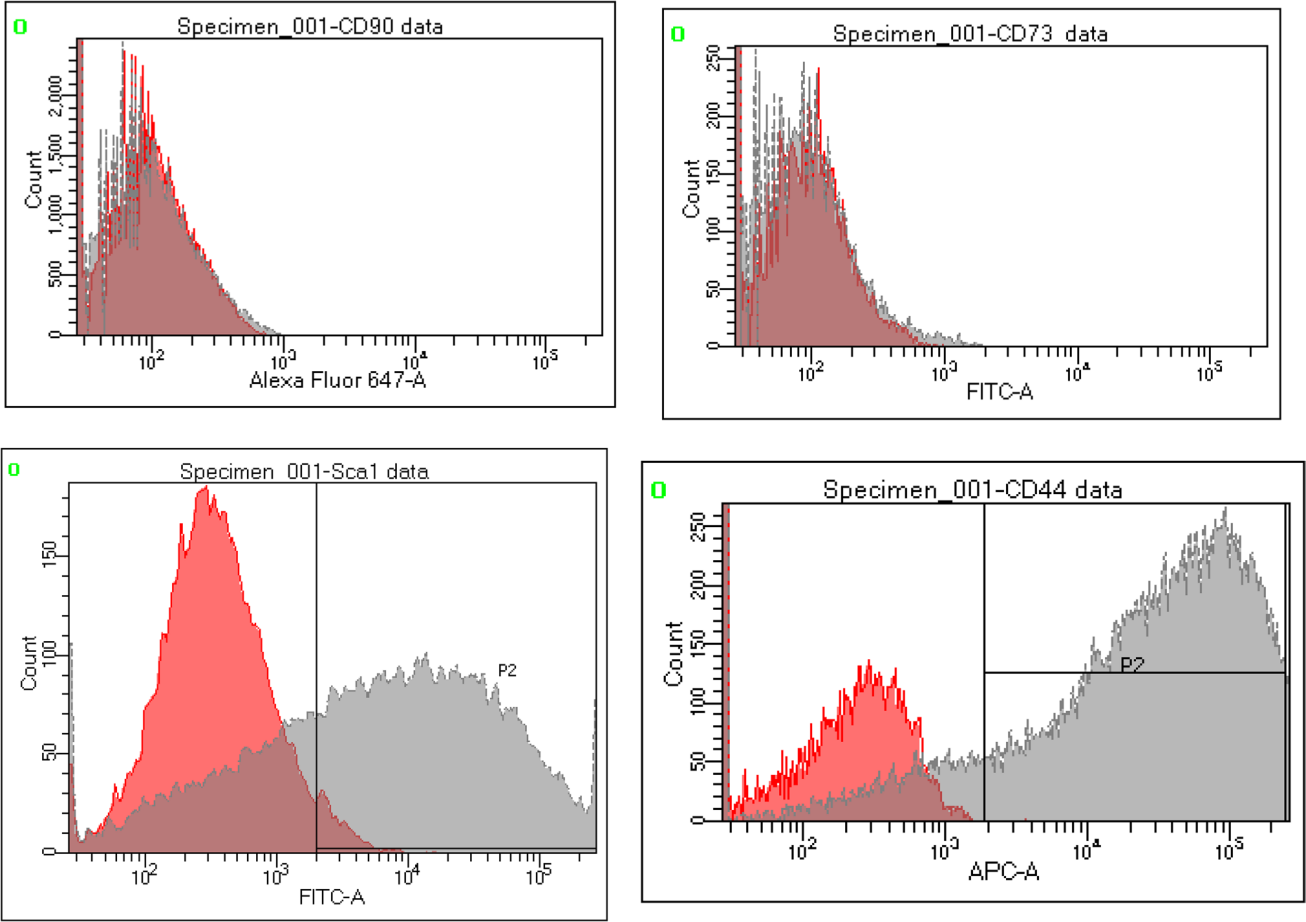
Characteristics of ADSCs extracted from mice inguinal adipose tissue. ADSCs were positive for the stromal cell markers, Sca-1 and CD44, and negative for the mesenchymal stem cell markers, CD73 and CD90

**Fig. 2:**
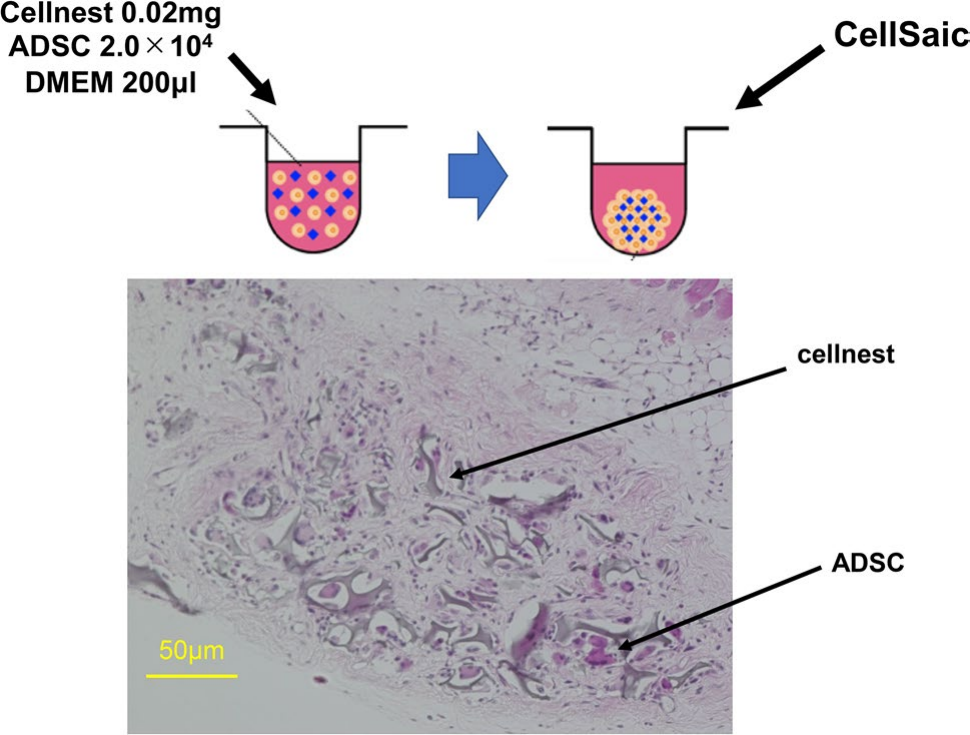
Synthesis of CellSaic by mixing Cellnest^®^ and ADSCs. CellSaic platforms containing ADSCs were prepared by mixing ADSCs (2.0 × 10^4^ cells) and Cellnest^®^ pieces (0.02 mg) in 200 μL Dulbecco’s modified Eagle’s medium (DMEM)

ADSCs extracted from C57BL/6N mice were positive for Sca-1 and CD44, which are markers for stromal cells, and negative for CD73 and CD90, which are markers for mesenchymal stem cells (Fig. 1). CellSaic containing ADSCs was synthesized as described (Fig. 2).

### Blood flow rate

**Fig. 3:**
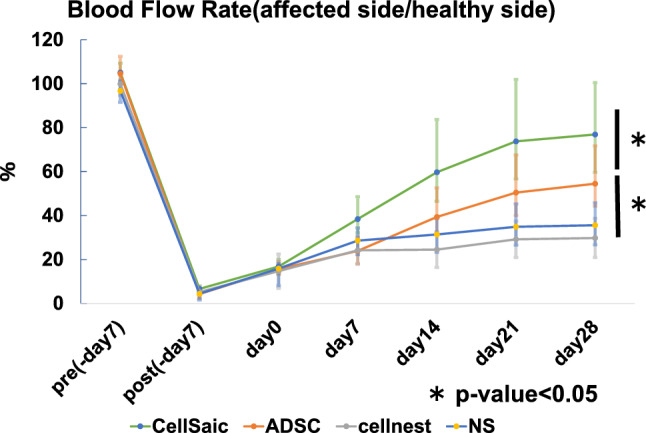
Improvement of blood flow in the affected lower limb. Compared with the control group, the treated side blood flow rate of the ADSC group showed significant improvement on the 28th day after administration. Compared with the ADSC group, the treated side blood flow rate of the CellSaic group showed significant improvement on the 28th day after administration

The blood flow rate (blood flow on the affected side/blood flow on healthy side × 100%) on the 28th day after administration was 76.9 ± 23.6% in the CellSaic group, 54.5 ± 17.2% in the ADSC group, 35.6 ± 10.2% in the saline group, and 29.8 ± 8.9% in the Cellnest group, as determined using a laser Doppler blood flow meter. Compared with the control group, the treated side blood flow rate of the ADSC group showed significant improvement on the 28th day after administration. Compared with the ADSC group, the treated side blood flow rate of the CellSaic group showed significant improvement on the 28th day after administration (Fig. 3).

### Number of vascular endothelial cells

**Fig. 4:**
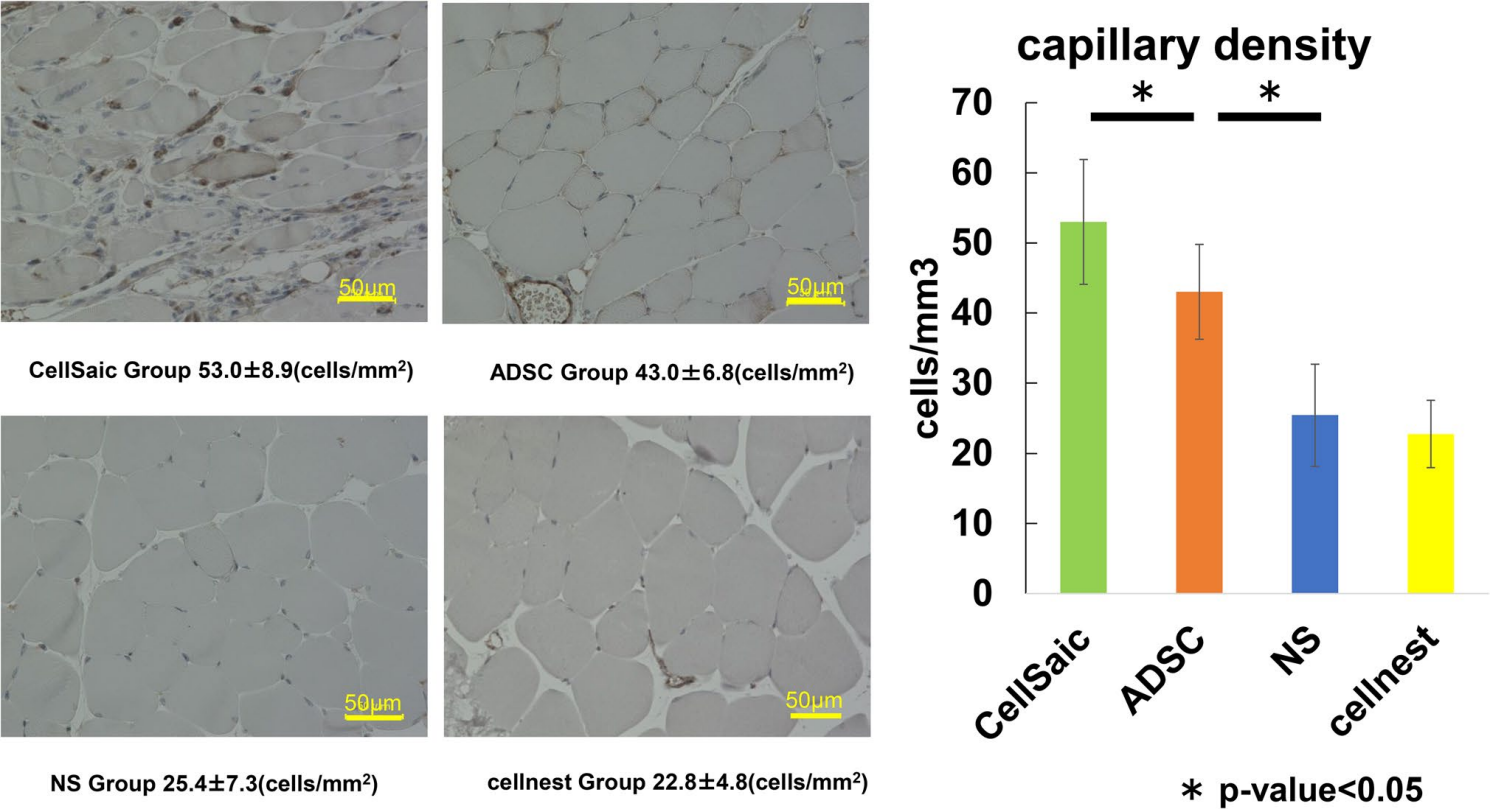
Enumeration of CD31-positive cells via immunostaining. The number of CD31-positive cells was significantly higher in the ADSC group than in the control group and was significantly higher in the CellSaic group than in the ADSC group

The number of CD31-positive cells was 53 ± 8.9 cells/mm^3^ in the CellSaic group, 43 ± 6.7 cells/mm^3^ in the ADSC group, 25.4 ± 7.2 cells/mm^3^ in the saline group, and 22.7 ± 4.8 cells/mm^3^ in the Cellnest group. The number of CD31-positive cells was significantly higher in the ADSC group than in the control group and was significantly higher in the CellSaic group than in the ADSC group (Fig. 4).

### Percentage of mature blood vessels

**Fig. 5:**
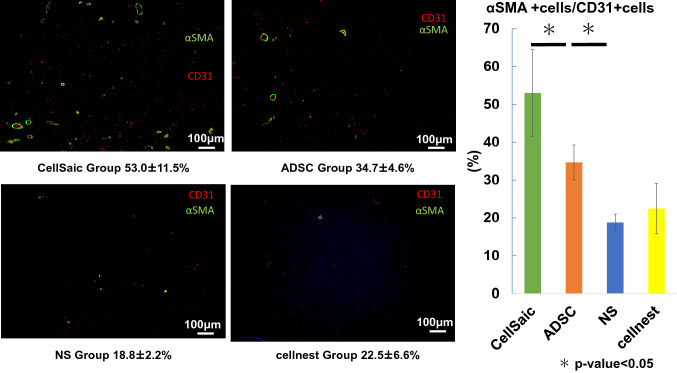
Evaluation of double-positive (CD31, αSMA) cells determined by fluorescent staining. The number of double-positive cells was significantly higher in the ADSC group than in the control group and was significantly higher in the CellSaic group than in the ADSC group

Fluorescent staining for the vascular endothelial cell marker CD31 and smooth muscle marker αSMA were performed, and the percentage of double-positive cells was evaluated. The percentage of double-positive cells was 53.0 ± 11.5% in the CellSaic group, 34.7 ± 4.6% in the ADSC group, 18.8 ± 2.2% in the saline group, and 22.4 ± 6.6% in the Cellnest group. The number of double-positive cells was significantly higher in the ADSC group than in the control group and was significantly higher in the CellSaic group than in the ADSC group (Fig. 5).

### Secreted angiogeneic factor

**Fig. 6:**
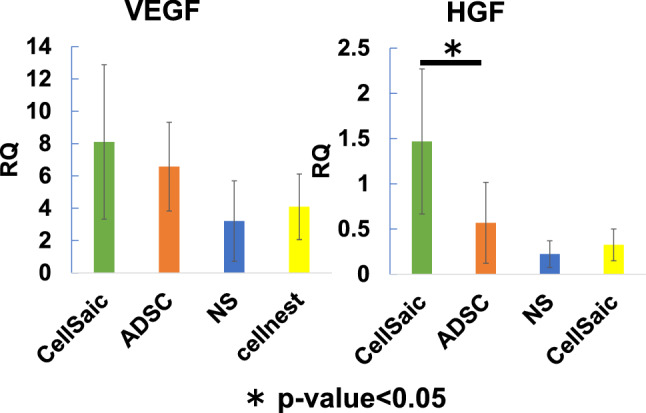
Angiogenic factors such as VEGF and HGF in muscle tissues analyzed using RT-PCR. There was no significant difference in VEGF expression between the CellSaic and ADSC groups. However, relative quantification (RQ) indicated significantly higher HGF expression in the CellSaic group than in the ADSC group

RT-PCR was performed on the 28th day after administration to evaluate VEGF and HGF expression. In terms of VEGF expression, there was no significant difference between the CellSaic and ADSC groups. However, relative quantification indicated significantly higher HGF expression in the CellSaic group than in the ADSC group (Fig. 6).

### Number of GFP-positive cells

**Fig. 7:**
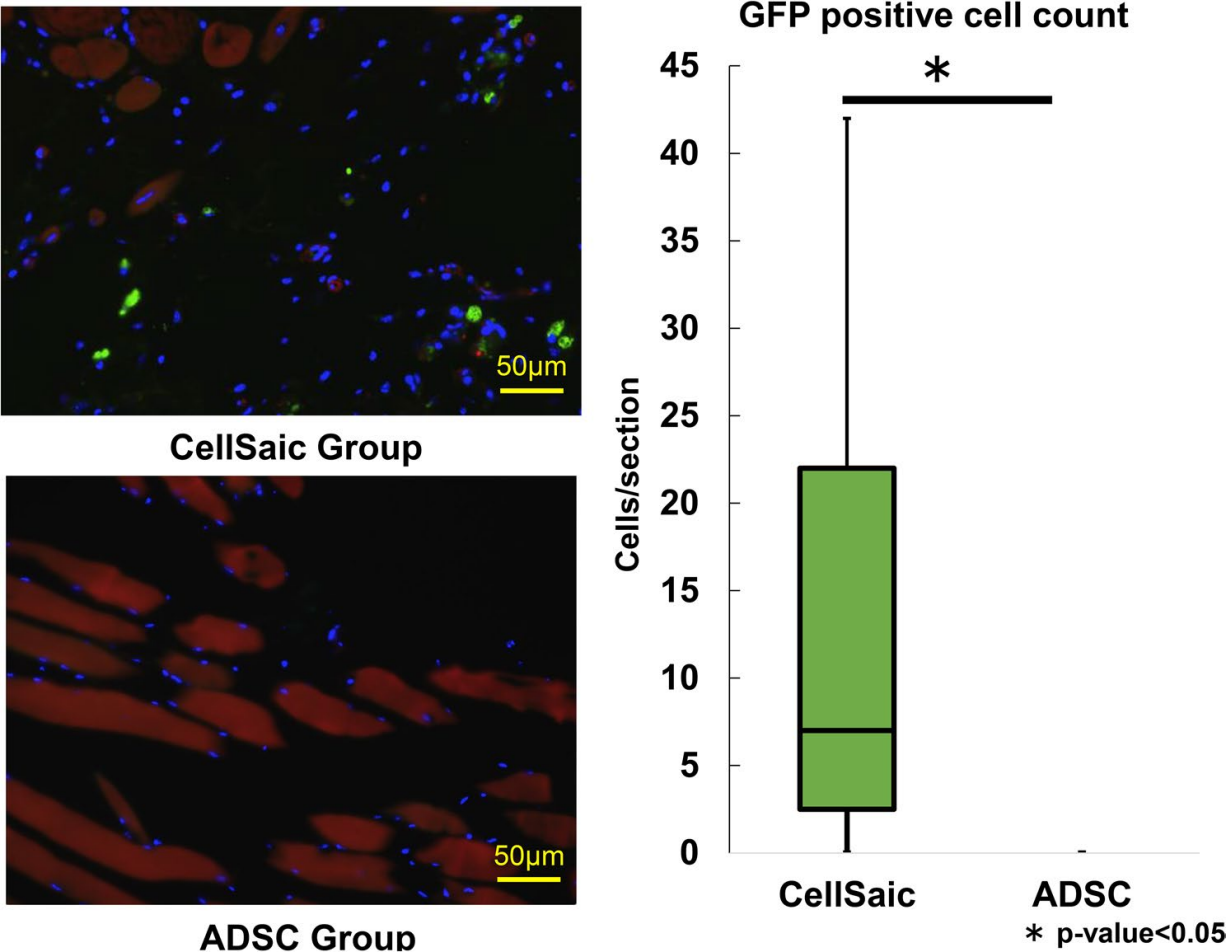
Evaluation of engraftment rate of administered cells using GFP-positive cells. No surviving cells were found in the ADSC group. However, in the CellSaic group, it was possible to confirm the presence of some engrafted cells

ADSCs extracted from GFP mice were used, and the number of GFP-positive cells in the muscle tissue collected on the 28th day after administration was evaluated. No surviving cells were found in the ADSC group. However, the presence of some engrafted cells could be confirmed in the CellSaic group (Fig. 7).

## Discussion

Most of ADSCs extracted from C57BL/6N mice were stromal cells and only a few mesenchymal stem cells. Since a previous study reported that stromal cells alone have a certain effect on angiogenesis [4], we used stromal cells. The results of the blood flow measurement suggested that CellSaic improved lower limb blood flow compared with ADSCs alone. The results of histological analyses suggested that both endothelial cells and mature blood vessels with smooth muscle tissue were significantly increased in the CellSaic group compared with those in the ADSC group. Moreover, compared with that in the ADSC group, the level of HGF, an angiogenic factor, was significantly increased in the CellSaic group. Although there was no significant difference, VEGF expression tended to be higher in the CellSaic group than in the ADSC group (*p* < 0.1); significant differences may be observed by increasing the sample size. In case of ADSCs extracted from GFP mice, no surviving cells were found in the ADSC group. However, in the CellSaic group, engrafted cells were detected at the transplanted site. This experiment demonstrated improved cell engraftment using CellSaic. Of note, the administered cells may have moved somewhere in the ADSC group. Given the fact that cells often do not stay at the planned administration site, we believe our observation of cells staying at the planned administration site for a longer period using CellSaic to be significant.

Cellnest, a CellSaic component, is enriched in the RGD sequence of the human type I collagen α-1 chain [3]. This RGD sequence is the major adhesive site of the integrin family proteins, which are adhesive proteins in the cell membranes [5]. Therefore, Cellnest exhibits strong cell adhesion and acts as an extracellular matrix to create appropriate intercellular spaces. Various cellular activities may be improved by the transmission of appropriate signals via integrins. CellSaic containing ADSCs exhibited better engraftment following transplantation than ADSCs alone, because central necrosis and anoikis were less likely to occur. Typically, nutrients are not distributed to the center of a cell mass, which leads to central necrosis [6, 7]. However, because CellSaic ensures appropriate intercellular spaces, blood vessels that nourish the cells grow into CellSaic from the host. Thus, CellSaic can avoid central necrosis following in vivo transplantation. In addition, cells that do not receive appropriate signals from the extracellular matrix are more likely to undergo anoikis, a type of apoptosis [8]. However, in the present study, Cellnest played a crucial role as an extracellular matrix and suppressed anoikis by forming CellSaic, leading to better cell engraftment following transplantation. Of note, the administered cells may receive extracellular matrix signals from the administered host. In fact, anoikis occurs in a short window between administration and extracellular matrix signal reception from the host [9].

In the present study, ADSCs collected from the mouse inguinal adipose tissues were used. ADSCs can differentiate into adipocytes and produce various adipocytokines following induction [10]. In particular, adiponectin shows anti-inflammatory and antioxidant properties, and it can suppress angiopathy and protect ischemic tissues via T-cadherin [11]. In addition, omentin, a type of adipocytokine, exerts angiogenic effects via AMPK–endothelial NO synthase signaling [12]. Therefore, ADSCs can secrete angiogenic factors, such as HGF, and can differentiate into adipocytes to secrete various adipocytokines involved in angiogenesis and tissue protection. This mechanism may promote blood flow in the lower limbs of mice with severe lower limb ischemia.

## Conclusion

Compared with the administration of ADSCs alone, that of CellSaic containing ADSCs promoted significant blood flow with enhanced engraftment in critical limb ischemia model mice.

## References

[1] Norgren L, Hiatt WR, Dormandy JA, Nehler MR, Harris KA, Fowkes FGR, TASC II Working Group. Inter-society consensus for the management of peripheral arterial disease (TASC II). J Vasc Surg 2007;45:S5–67. 10.1016/j.jvs.2006.12.037 17223489

[2] Cooke JP, Losordo DW (2015). Modulating the vascular response to limb ischemia: angiogenic and cell therapies. Circ Res.

[3] Nakamura K, Iwazawa R, Yoshioka Y (2016). Introduction to a new cell transplantation platform via recombinant peptide petaloid pieces and its application to islet transplantation with mesenchymal stem cells. Transpl Int.

[4] Nakagami H, Maeda K, Morishita R, Iguchi S, Nishikawa T, Takami Y, Kikuchi Y, Saito Y, Tamai K, Ogihara T, Kaneda Y (2005). Novel autologous cell therapy in ischemic limb disease through growth factor secretion by cultured adipose tissue–derived stromal cells. Arterioscler Thromb Vasc Biol.

[5] Kapp TG, Rechenmacher F, Neubauer S, Maltsev OV, Cavalcanti-Adam EA, Zarka R, Reuning U, Notni J, Wester HJ, Mas-Moruno C, Spatz J, Geiger B, Kessler H (2017). A comprehensive evaluation of the activity and selectivity profile of ligands for RGD-binding integrins. Sci Rep.

[6] Shirakawa K, Tsuda H, Heike Y, Kato K, Asada R, Inomata M, Sasaki H, Kasumi F, Yoshimoto M, Iwanaga T, Konishi F, Terada M, Wakasugi H (2001). Absence of endothelial cells, central necrosis, and fibrosis are associated with aggressive inflammatory breast cancer. Cancer Res.

[7] Mueller-Klieser W (1997). Three-dimensional cell cultures: from molecular mechanisms to clinical applications. Am J Physiol.

[8] Attwell S, Roskelley C, Dedhar S (2000). The integrin-linked kinase (ILK) suppresses anoikis. Oncogene.

[9] Zvibel I, Smets F, Soriano H (2002). Anoikis: roadblock to cell transplantation?. Cell Transpl.

[10] Maeda K, Okubo K, Shimomura I, Mizuno K, Matsuzawa Y, Matsubara K (1997). Analysis of an expression profile of genes in the human adipose tissue. Gene.

[11] Parker-Duffen JL, Nakamura K, Silver M, Kikuchi R, Tigges U, Yoshida S, Denzel MS, Ranscht B, Walsh K (2013). T-cadherin is essential for adiponectin-mediated revascularization. J Biol Chem.

[12] Maruyama S, Shibata R, Kikuchi R, Izumiya Y, Rokutanda T, Araki S, Kataoka Y, Ohashi K, Daida H, Kihara S, Ogawa H, Murohara T, Ouchi N (2012). Fat-derived factor omentin stimulates endothelial cell function and ischemia-induced revascularization via endothelial nitric oxide synthase-dependent mechanism. J Biol Chem.

